# Of Mice and Men: Comparative Analysis of Neuro-Inflammatory Mechanisms in Human and Mouse Using Cause-and-Effect Models

**DOI:** 10.3233/JAD-170255

**Published:** 2017-07-29

**Authors:** Alpha Tom Kodamullil, Anandhi Iyappan, Reagon Karki, Sumit Madan, Erfan Younesi, Martin Hofmann-Apitius

**Affiliations:** a Department of Bioinformatics, Fraunhofer Institute for Algorithms and Scientific Computing, Sankt Augustin, Germany; b Rheinische Friedrich-Wilhelms-Universität Bonn, Bonn-Aachen International Center for IT, Bonn, Germany

**Keywords:** Alzheimer’s disease, human, mice, neuroinflammation

## Abstract

Perturbance in inflammatory pathways have been identified as one of the major factors which leads to neurodegenerative diseases (NDD). Owing to the limited access of human brain tissues and the immense complexity of the brain, animal models, specifically mouse models, play a key role in advancing the NDD field. However, many of these mouse models fail to reproduce the clinical manifestations and end points of the disease. NDD drugs, which passed the efficacy test in mice, were repeatedly not successful in clinical trials. There are numerous studies which are supporting and opposing the applicability of mouse models in neuroinflammation and NDD. In this paper, we assessed to what extend a mouse can mimic the cellular and molecular interactions in humans at a mechanism level. Based on our mechanistic modeling approach, we investigate the failure of a neuroinflammation targeted drug in the late phases of clinical trials based on the comparative analyses between the two species.

## INTRODUCTION

Neuroinflammation is the hallmark of almost all neurodegenerative diseases (NDDs) including Alzheimer’s disease (AD) [1]. Aggregated amyloid-β (Aβ) peptides are believed to trigger the innate immune response through microglial and astroglial cells, which may lead to exacerbation of the disease [2]. Studies on early stage of AD as well as rodent models suggest that immune actions alone are sufficient to cause AD-like pathology and can precede tau and amyloid pathology in the brain [3]. As a consequence, neuroinflammation in AD has been proposed as an attractive target for therapeutic modulation and prevention [4]. Modulation of neuroinflammation for drug target- or biomarker identification requires extensive use of rodent models of AD to study molecular drivers of inflammation and various disease phenotypes associated to it [5]. Despite the availability of different mouse models representing APP mutations or tauopathy, the results of neuroinflammation modulation in these models have been divergent, suggesting that currently available mouse models do not accurately reflect human AD pathology [6]. For instance, conventional transgenic models of AD, which are routinely used for preclinical studies, have been shown to incompletely mirror the inflammatory response seen in AD human brains [7]. Work of Seok et al. in 2013 that reported on poor recapitulation of genomic responses of human inflammatory diseases in mouse models [8] stimulated the old debate [9] as to whether animal models can reliably inform human diseases. However, statistical re-evaluation of Seok et al. results by Takao and Miyakawa in 2014 suggested that correlations between gene expression patterns from mouse models and human conditions were stronger than reported originally [10]. Warren et al. re-confirmed essential differences between these two species at molecular level by showing that mouse models mimicked only 12% of the genes deregulated in human conditions [11]. Beside these inter-species differences between inflammatory responses in human and mouse at molecular level, a similar significant difference also exists at the brain anatomical level, including greater size, higher lobular organization, more developed sulci and gyri, and larger amount of white matter in the human brain [12]. Importantly, such anatomical differences have underlying molecular correlates as demonstrated by the atlas of the mid-gestational human brain [13].

Taking into account 65 million years of inter-species evolutionary divergence, it is not surprising that there are also significant discrepancies in both innate and adaptive immunity between human and mouse, including differences in immune receptors, cell types, and signaling pathways [14]. Such substantial inter-species differences can have considerable impact on drug discovery and development efforts. In fact, biomedical research has long relied on experimentation in mice to investigate human diseases and evaluate drug candidates. The value of animal models in drug discovery and development cannot be overstated even though the failure of the clinical trials can be attributed to other factors like poor design of the trials (wrong dose or endpoint), different genetic make-up among patients, and so many other logistic issues. However, the high rate of drug failures in general start right from selection of the correct molecule in pre-clinical studies and recent failures of AD therapies in phase III of clinical trials, in particular, again point to the fact that inter-species discrepancies at all biological levels should be seriously taken into consideration before proceeding to expensive clinical trials. Computational systems models can facilitate this task by gathering both experimental data and published knowledge, standardizing this information, integrating them across various biological scales, and representing this species-specific information in the form of consolidated cause-and-effect digital models. We have already shown the value of such approach for identification of disease-specific pathways in AD as compared to normal bioprocesses in the human brain [15], and Pappalardo et al. built computational model in immune system, which predicts how immune system activates in different conditions [16]. Motivated by these results, we sought to systematically model and mechanistically compare neuroinflammatory pathways specific to microglia, astrocytes, macrophages, and neurons between human and mouse in the context of AD. Biological Expression Language (BEL) [17] was used to build cause-and-effect computable models of neuroinflammation for both human and mouse based on published knowledge in the biomedical literature. Comparison of human and mouse models were performed at structural and functional levels, with the aim of answering the question, whether our current knowledge about neuroinflammation in mouse and human allows us to speak about a “functional equivalence” between these two species. In this work, we present the species-specific models and discuss which functional elements of neuroinflammation are similar and which elements are different between the two species. We also demonstrate, how the modeling approach can be used to explain— at a mechanistic level— the failure of translation from preclinical to clinical phase using a given drug in clinical phase III.

## METHODS

### Corpus selection and construction of neuroinflammation models specific for mouse and human

**Fig.1:**
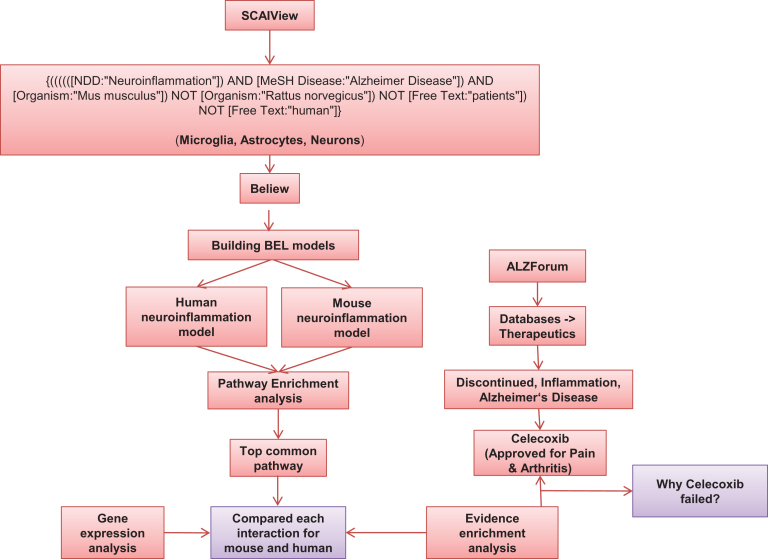
Workflow used for construction of models and their analysis.

Based on the workflow illustrated in Fig. 1, we have built neuroinflammation BEL models for human and mouse by extracting knowledge from literature, which are specific to three cell types in neurons: astrocytes, microglia, and macrophages; as they are actively involved in neuroinflammation.

To build specific models for human and mouse, we have generated relevant literature corpora using SCAIView [18], a text mining tool developed in Fraunhofer SCAI. We specifically selected articles, which are specific to neuroinflammation in human as well as in mouse. We retrieved 189 documents for mouse using the following query: {*((((([NDD:“Neuroinflammation”]) AND [MeSH Disease:“Alzheimer Disease”]) AND [Organism:“Mus musculus”]) NOT [Organism:“Rattus norvegicus”]) NOT [Free Text:“patients”]) NOT [Free Text:“human”]*}. Out of 189 documents, we manually filtered 173 articles which were further incorporated into the ‘Mouse neuroinflammation model’. Similarly, 309 articles were harvested for human using the query {*(((((((((([NDD:“Neuroinflammation”]) AND [MeSH Disease:“Alzheimer Disease”]) AND [Organism:“Homo sapiens”]) NOT [Organism:“Mus musculus”]) NOT [Organism:“Rattus rattus”]) NOT [Free Text:“mice”]) NOT [Free Text:“mouse”]) NOT [Free Text:“murine”]) NOT [Organism:“Rattus sordidus”]) NOT [Free Text:“rat”]) NOT [Free Text:“rodent”]*} and 152 articles were used to build the ‘Human neuroinflammation model’. The selected articles were subjected to analysis of causal and correlative relationships using the the BEL Information Extraction workflow (BELIEF) [19], a semi-automatic system that identifies cause-and-effect relationships in scientific text. The statements proposed by BELIEF were semi-automatically extracted, converted into BEL statements and further curated manually to build the neuroinflammation BEL models.

### Comparison of mouse and human neuroinflammation models

#### Comparison based on interactions from species-specific BEL models

A systematic comparative analysis was done based on the molecular involvement of genes, bioprocesses, and pathways. The BEL models were used to compare pathways and we identified shared as well as unique pathways, based on BEL statements and entities. In addition to this, we have done a pathway enrichment analysis using DAVID for human and mouse, by giving the complete gene set as input from each of the models (Supplementary Table 1) and compared the most enriched pathways (Supplementary Table 2). To identify the consistency between mouse and human interactions, we have done an additional manual evidence enrichment from the literature (Supplementary Table 3).

#### Comparison based on gene expression data for genes from species-specific models

To identify the concordance at the gene expression level between the two species, human and mouse, we have analyzed the representation of expressed genes in our models using gene expression datasets from Gene Expression Omnibus (GEO) [20]. We have analyzed 6 different gene expression datasets (Mouse: GSE35338, GSE74615, GSE74995 and Human: GSE26927, GSE45880, GSE59671) to support the findings of our study, all of which were related to neuroinflammation. GSE35338 contains expression data from astrocytes of mice where inflammation is induced by lipopolysaccharide treatment. Similarly, GSE74615 provides expression values from astroglia and microglia of transgenic mice, whereas GSE74995 has expression profiles of cortical tissue of AD transgenic mice. GSE45880 contains cytokine-induced expression profiles of human cerebral endothelial cell. GSE26927 contains expression data of males and females from different NDD patients, of which we considered only AD-related datasets. Lastly, GSE59671 contains expression values of RNAs of human smooth muscle cells treated with celecoxib and rofecoxib. All datasets were analyzed using the GEO2R tool provided by GEO [21].

## RESULTS

### Differential analysis of human and mouse neuroinflammatory pathways at the molecular level using cause-effect models

The neuroinflammation model for human consists of 884 BEL statements comprising 671 nodes and 1,224 edges extracted from 152 articles. Likewise, the mouse neuroinflammation model consists of 1,016 nodes and 1,939 edges specific to mouse, supported by 1,395 BEL statements from 173 articles. Even though we have a higher number of articles that discuss neuroinflammation in humans than mouse, we found that biological entities and relationships are in fact highly redundant among human specific articles. However, we could integrate more BEL statements and a larger variety of entities in the mouse model, as a higher number of novel molecular interactions have been studied in transgenic mouse experiments. For example, in case of App (Amyloid precursor protein), there are 155 transgenic mouse models, which have been generated for studies on amyloid biology [22].

In order to find shared and unique pathways between mouse and human, we have done a differential analysis between the two models. Gene set enrichment analysis was performed on genes in each model using the DAVID tool [23] to identify the most enriched pathways in both models. We retrieved 42 pathways in the human model and 29 pathways in the mouse model, of which 19 pathways were unique to human model and 24 pathways were found common between the two (Supplementary Table 2).

**Fig.2:**
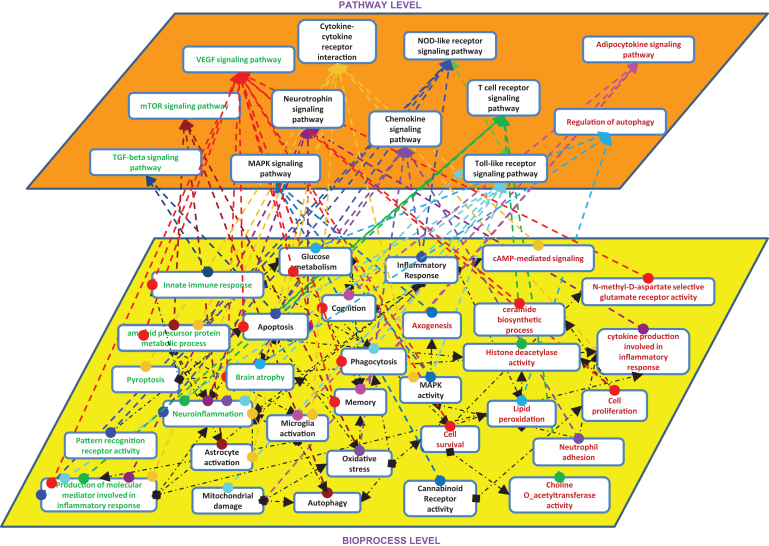
Shared pathways and bioprocess between mouse and human. Entities present in both models are in black color, enriched pathways in mouse are in RED color, enriched pathways in human are in GREEN color.

Among the 19 unique pathways in the human neuroinflammation model, we found VEGF signaling pathway and mTOR signaling pathway as the two top ranked pathways. In the case of mouse models, we found only 6 unique pathways but they were not specific to neuroinflammation. Based on these findings, we linked the bioprocesses corresponding to each pathway from the neuroinflammation models (Fig. 2). Despite shared pathways between the species, we found differential molecular patterns at the level of bioprocesses. For instance, bioprocesses like pyropotosis and pattern recognition receptor activity are better represented in the human model than the mouse model. However, when we extend these bioprocesses to pathways like cytokine-cytokine receptor interaction and Nod-like receptor signaling pathway, we can see that some parts of these pathways (e.g., inflammatory response or astrocyte activation) are well investigated in mouse experiments. Therefore, the resolution of mechanistic knowledge at the level of bioprocesses is higher in the mouse model than in the human model. At the abstraction level of canonical pathways, there are more commonalities between the two species, than at the level of underlying “cause-and-effect” mechanisms.

**Fig.3:**
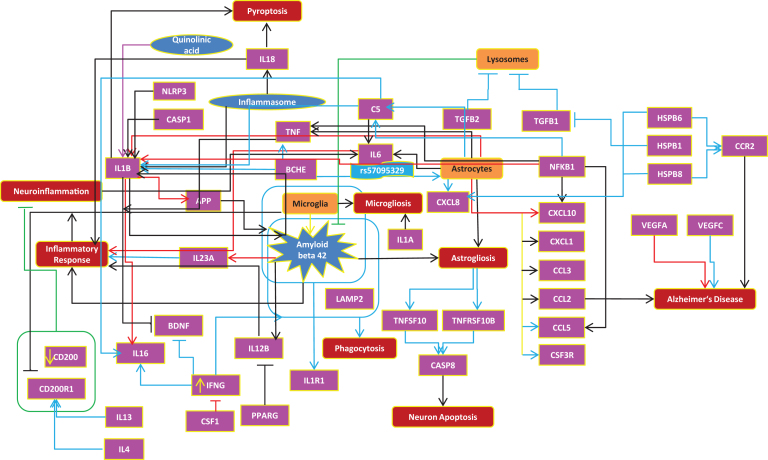
Cytokine-cytokine receptor interaction (human and mouse). BLACK color indicates interactions are consistent with human, RED color depicts contradictory interactions in mouse compared to human, and BLUE arrows show the interactions found only in human.

We also sought to identify to which extent the mouse model can represent human interactions at the molecular level. For this purpose, we investigated in more details the top common pathway (from DAVID analysis) between the two species; that is, cytokine-cytokine receptor interaction pathway. As shown in Fig. 3, there are 73 interactions in this pathway, which were represented in the human neuroinflammation model. Out of these 73 interactions, 33 interactions are protein-protein interactions at molecular level and 40 interactions among proteins, cell types, and bioprocesses, which are at cellular and bioprocess levels.

Furthermore, we have checked additional literature for more evidences on each interaction in the human cytokine-cytokine pathway and compared these against the mouse model to see how many of the molecular and cellular interactions proven in humans are already reflected in the mouse model. At the molecular level, we found that 27% of cytokine interactions are similar in both species, 15% of interactions in mouse are found to be contradictory (opposite direction) to human, and 58% of interactions were found only in human or in other words, 58% of these human interactions are not proven with mouse experiments (Supplementary Table 4).

These numbers should be interpreted with regard to the bias of our knowledge repertoire toward molecular research and publication on mouse and human experiments. However, within these limits, it can be observed that our current knowledge in the domain of cytokine pathway not only reflects the contradictions in molecular interactions between human and mouse, but also misses many comparable human interactions in the mouse model.

At the cellular level, based on the interactions among cell types (microglia and astrocytes), bioprocesses, and proteins, we found a relatively higher similarity between the two species. 62% of the interactions were similar between the species, 28% of interactions were found only in human, and 10% of interactions were found to be contradicting in human and mouse.

In addition to the above comparison and to support our findings from model comparison, we have done an overall analysis on the availability of gene expression data for each species (Supplementary Table 5). We found 13 experiments with the topic of neuroinflammation in human and 32 experiments in mouse or rat (Supplementary Table 5). From these experiments, we have further selected GSE74615, GSE35338, and GSE74995 for mouse, as these experiments were performed using brain-specific tissues like astrocytes, microglia, and cortex. Based on these experimental data, we have investigated how many cytokine interactions in our model are supported by independent transcriptome data. Similarly, we have conducted gene expression analysis for human brain tissues using GSE26927 (entorhinal cortex) and GSE45880 (cerebral endothelial cell line) datasets. Since these experiments have not used the same tissues in human and mouse, a direct comparison between the two species is not possible, considering the fact that genes can be expressed differently in different tissues and regions. Excluding tissue specificity, expression patterns for 31% of genes in both species were the same, while 14% of genes from Fig. 3 were significantly expressed only in human and 19% of genes were expressed only in mouse. 17% of genes were identified to be inconsistent (same gene is shown as up- and downregulated) within the species and 7% of genes showed to have contradictions between the species. 12% of genes were found to be statistically non-significant (*p*-value >0.05) in both species (Supplementary Table 6).

### Analysis of failure of drugs on the basis of translation between species

Based on the approach suggested by Younesi and Hofmann-Apitius for translational validation of disease models [24], we aimed to identify the extent to which the mouse model can translate into the human biological interactions by including the mode-of-action of drugs within the mechanistic model. For this purpose, we performed an analysis based on failed drugs in AD that specifically were targeted against neuroinflammation as these drugs proved to work in mouse models in pre-clinical development, but failed in human during clinical trials.

Accordingly, drugs which have failed in AD clinical trials were collected from the Therapeutics database of AlzForum [25], using the following search query: Food and Drug Administration (FDA) status – discontinued, Target Type – Inflammation, Therapy Type – All, Condition – Alzheimer’s disease and Mild cognitive impairment.

We were able to retrieve 8 failed or discontinued AD therapeutics, out of which, celecoxib which is approved for pain and arthritis was selected for analysis [26, 27]. The rationale behind this selection is that there were many lines of evidence supporting the role of comorbidity association between rheumatoid arthritis and AD [28–30], and points to a likely shared mechanism at the molecular and cellular levels. Thus, we performed mechanistic analysis around the targets of celecoxib, both for human and mouse, in order to find probable mechanistic differences in the translation of interactions between the two species which might have led to the failure of celecoxib in AD.

Celecoxib has two main targets namely: PTGS2 (prostaglandin-endoperoxide synthase 2 (prostaglandin G/H synthase and cyclooxygenase)) and PDPK1 (3-phosphoinositide dependent protein kinase 1).

According to transgenic mouse experiments, in normal conditions Pdpk1 increases the activity of Il4 and Ins [31] and also phosphorylates Gsk3b and Akt1 [32]. Pdpk1 also inhibits Ccr2, M1 macrophages, and insulin resistance [31]. Similarly, Ptgs2 increases Aβ peptides [33, 34], which further increases Tnf and Nfkb1 [35]. Nfkb1 increases Ptgs2 forming a self-regulatory network [36] leading to an increase in Aβ peptide aggregation and an increase in inflammation due to increased activation of Tnf. Based on the above-mentioned interactions deduced from mouse experiments, inhibition of PTGS2 and PDPK1 with administration of celecoxib seems to be a good tactic in treating neuroinflammation. Here are some of the positive effects of celecoxib in case of AD and neuroinflammation (mainly based on mouse experiments and few supportive evidence from human experiments):

• Celecoxib increases M1-macrophage^1^ and Ccr2 and thereby increases phagocytosis and Aβ clearance in mouse models respectively [31].
• Furthermore, Pdpk1 inhibition of Gsk3b phosphorylation by celecoxib prevents the formation of neurofibrillary tangles through phosphorylated tau (Mapt).

However, we have extended our investigation very specific to the celecoxib interactions particularly in the context of AD on the basis of mouse models.

We have also deduced the perturbation caused in normal physiological brain pathway in human upon administration of celecoxib. The following lines of evidence provide, at mechanism level, explanatory insight why celecoxib could not work in humans as expected in mouse:

• In the case of Pdpk1 inhibition, phosphorylation of Akt1 can be reduced which may further increase the phosphorylation of Tsc2. According to Shang et al., it was proposed that phosphorylation of threonine at position 1462 of Tsc2, a target of Akt1, is increased in AD [37], and supported by the finding that Tuberin (TSC2) was hyperphosphorylated at Thr1462 in postmortem frontal cortex tissue of both AD and PD/DLB patients [38].
• Hyperphosphorylated Tsc2 hyperactivates Mtor through Rheb which reduces autophagy [39–41]. If autophagy is reduced, then it will lead to increased amyloid deposition.
• Also, a very recent paper by Oddo et al. stated that decreased mTOR activity may be necessary to decrease BACE1 and reduce Aβ generation in AD from mouse experiments [41]. Therefore, as a result of reduction in Akt1 activity upon celecoxib administration, Mtor hyperactivates and leads to increased amyloid deposition.
• Celecoxib increases IL-4 (which is anti-inflammatory protein) inhibition which causes inflammation [43]. Similarly, inhibition of Pdpk1 by celecoxib might cause increase in insulin resistance through inhibition of Ins. Insulin resistance is proposed to be a risk factor for AD [44].
• Upon celecoxib administration, it inhibits PDPK1 (PDK1) which reduces the phosphorylation of AMPK (PRKAA1) (which in normal physiology reduces MTOR activation) leading to the inhibition of autophagy. This leads to decrease in Aβ clearance [45, 46]. This disease mechanism has been described already in detail by Kodamullil et al. [15].

Based on the above reports (evidence which support the usage of celecoxib with mouse experiments and evidence how the normal physiological mechanism in human brain is perturbed upon administration of celecoxib), we can conclude that even though celecoxib modulates MTOR toward neuronal protection to limit the toxicity of Aβ and consequently neuroinflammation in AD, we may also require targeting TSC2, AKT, and AMPK simultaneously. It is noteworthy at this point that AD and neuroinflammation in humans are so complicated that it appears unlikely that an experimental design based on a specific transgenic mouse manipulated for a single gene allows us to expose all the interlinked mechanisms.

**Fig.4:**
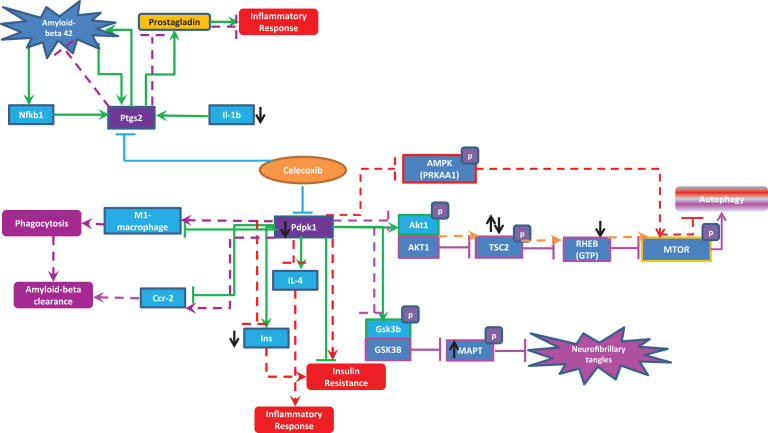
Celecoxib interactions in human and mouse. The green lines represent the normal brain interaction of celecoxib targets (Pdpk1 and Ptgs2) with other entities proposed in mouse experiments. The red dotted lines indicate how normal pathways might be perturbed upon administration of celecoxib, which could lead to severity of neuroinflammation and AD. The purple dotted lines are the beneficial effects of celecoxib in the context of AD and neuroinflammation. The black up and down arrows represents the expression of genes (upregulation and downregulation) upon celecoxib administration.

To validate the celecoxib interactions shown in Fig. 4, we have done gene expression analysis using the gene expression experiment GSE59671, which is the only dataset available related to our research context, even though the cell line used was human aortic smooth muscle cells (3F1243) pre-treated with rofecoxib (500 nM) or celecoxib (500 nM) (Supplementary Table 5). As seen in Fig. 4, the expression data supports the reduction in levels of INS, Il1b, and RHEB, and increase in TSC2 although there are inconsistencies about increased MAPT in human upon celecoxib administration.

## DISCUSSION

Mouse models are extensively used in biomedical research mainly to understand the etiology of the disease. Complex diseases like AD may involve several simultaneous alterations in molecular and processual activities, including neuroinflammation, aggregation of Aβ peptides, or tau phosphorylation, which are likely to contribute to pathophysiology. In this paper, we have compared the mouse and human at molecular, cellular, and pathway levels to shed light on mechanistic differences with important implications for translation outcomes. Mechanistic modelling specific to species allows us to “embed” and “represent” similarities and differences in innate immunity which can lead to the development of “conflictious information detection engine”. It is important to note that our analysis is purely based on the research and publication bias in mouse and human experiments as many mouse experiments are mainly focused on particular explorative areas, and experiments with human tissues are also concentrated on limited areas of disease mechanism. We found that mouse experiments often reveal new molecular interactions between different entities that are not observed or reported in human experiments. Differential analysis of mouse and human model for neuroinflammation shows that mouse and human differ at the molecular and cellular levels, but have more similarities at the pathway levels as numbers indicate. More explicitly, the underlying molecular patterns which lead to a particular bioprocess differ between the two species. This finding implies that although the two species share some similarities at the cellular or pathway level, the pattern of molecular interactions that form, govern, and regulate those pathways is substantially different between mouse and human.

It is notable that mouse models have provided significant insights into many disease areas like cancer; acute promyelocytic leukemia. However, recent drug failures in the area of neurodegeneration have put a question mark behind the extent to which mouse models have been used in preclinical drug discovery and to what extent transgenic mice mimic human brain pathophysiology mechanisms. Pathophysiology mechanisms are likely to act together and they seem to be organized in a temporal cascade of events that ultimately result in a severe disease phenotype. Experiments with single gene knock-out in mice can reveal only minor aspects of the disease perturbations and do not usually allow us to decipher the full complexity of the mechanisms underlying the disease. For example, even though high amounts of Aβ are observed in APP knock-in mice carrying the Swedish mutations, these mice do not produce amyloid plaques [47]. On the other hand, human APP K670N-M671L (APPSw), which have amyloid deposition and behavioral deficits, do not exhibit any neuronal loss [48]. This points to the fact that each strain of mouse results in various phenotypes and do not represent the main clinical outcome. This emphasizes the need to do systematic comparisons between the model organism (and factual findings in mice and rats) and human. Additionally, development of various mouse models should also consider the absence of key functional human genotypes (Apoe 3,4) in animal models. If the above hypothesis regarding systematic differences at molecular level among species holds true, then the expectation is to observe different or multipoint translational outcomes in human compared to mouse. The most striking case of a different outcome happens when a drug fails and the most common case of a multipoint outcome is serendipitous effect of a drug on an unexpected biology. Even in the case that drug candidate successfully hits the pathology, the subsequent side effects clearly show the underlying mechanistic differences between human and mouse. Comparative analysis of the mode-of-action of celecoxib in the neuroinflammatory pathway between human and mouse at the high-resolution molecular level demonstrates that perhaps target studies ignore human unique pathways and the underlying unique mechanisms. It was found that many off-target interactions that could occur in human were not considered in the mouse experiments. The fact that mimicking human disease pathology in mice using a chemical agent or a single gene is purely correlative and supports the notion that it is crucial to take the fundamental mechanistic differences between mouse and human into consideration when attempting to translate preclinical findings to clinical trials. This is not intended to criticize the use of mouse models (considering the fact that failure of clinical trials are not solely associated with mouse models, rather also to differences in patient level, drug dosage, etc.) but rather to point out the repetitive failure of clinical trials in AD and neuroinflammation indications. Therefore, it is time to rethink about the caveats inherent with the mouse model experiments. The construction and simulation of computable cause-and-effect models of disease pathology can greatly increase the probability of translational success. The computable cause-and-effect modeling approach described in this work can be complemented with a systems biology simulation at systems level. Such in-silico models can effectively contribute to well-informed design of *in vivo* mouse models by predicting the expected and unexpected outcomes compared to human conditions. We foresee that with the advent of big biomedical data and growth of published knowledge, disease-specific computable models will play an important role in drug discovery and biomarker identification for clinical applications.

## Supplementary Material

Supplementary Table 1 Table which included the genes from both mouse and human, which are submitted to DAVID tool for functional annotation of genes for retrieving pathways.

Supplementary Table 2 Canonical pathways which are enriched with the genes from the species specific models.

Supplementary Table 3 Supporting evidences and comparison of interactions from mouse in humans for Fig. 3.

Supplementary Table 4 Percentage of translation in human and mouse interactions.

Supplementary Table 5 Information regarding Gene expression datasets used in analysis.

Supplementary Table 6 Gene expression of genes from Figs. 3 and 4.

## Footnotes

^1^ The authors are aware of the fact that M1/ M2 nomenclature of macrophages is most likely obsolete or needs new interpretation as pointed out by Xue J. et al. 2014 (Immunity. 2014 Feb 20; 40(2): 274–288), but we stick to the “M1 object” in our models, as defined processes and properties have been associated with this object are derived from other researchers.
